# Stereotactic radiosurgery of benign brain tumors in elderly patients: evaluation of outcome and toxicity

**DOI:** 10.1186/s13014-020-01714-0

**Published:** 2020-12-09

**Authors:** Daniel Rueß, Vera Weyer, Juman Tutunji, Stefan Grau, Martin Kocher, Mauritius Hoevels, Harald Treuer, Christian Baues, Maximilian I. Ruge

**Affiliations:** 1 Department of Stereotaxy and Functional Neurosurgery, Centre of Neurosurgery, Medical Faculty and University Hospital Cologne, University of Cologne, Kerpener Straße 62, 50937 Cologne, Germany; 2 Department of Neurosurgery, Centre of Neurosurgery, Faculty of Medicine, University of Cologne, Kerpener Straße 62, 50937 Cologne, Germany; 3 Institute of Radiation Oncology, Faculty of Medicine, University of Cologne, Kerpener Straße 62, 50937 Cologne, Germany

**Keywords:** Radiosurgery, Vestibular schwannoma, Meningioma, Elderly patients

## Abstract

**Background:**

Stereotactic radiosurgery (SRS) is widely accepted as a therapeutic option for meningiomas (M) and vestibular schwannomas (VS). However, data on outcome and toxicity in the elderly population have rarely been reported in detail.

**Methods:**

All patients aged ≥ 65 years with M or VS who underwent single fraction SRS were included. Patient data were analyzed in terms of clinical tumor control and incidence of early and late treatment related complications, which were graded according to the Common Terminology Criteria for Adverse Events (CTCAE),

**Results:**

We identified 245 patients with benign brain tumors (129 M and 116 VS, median tumor volume 2.9 ml, range 0.1–28). The median age was 71 years (range 65–86) and the mean follow-up times were 42 months (range 2–181). Tumors were irradiated with a median dose of 12.4 Gy. Actuarial clinical and radiological tumor control rates at 2, 5, and 10 years after SRS were 98%, 93%, and 88%, respectively. Recurrent tumors after previous treatment had a higher probability of post-radiosurgical progression (*p* < 0.001). Permanent toxicity (CTCAE I/II) were noted in 5.7%. No severe adverse events were observed during early and late follow up, although patients > 70 years had a slightly higher risk for toxicity (*p* = 0.027). The presence and extent of co-morbidities had no significant influence on local tumor control or toxicity.

**Conclusion:**

SRS provides favorable tumor control with low risk for treatment-related severe complications. Thus, SRS should always be considered as treatment option for benign intracranial tumors (meningiomas, schwannomas), especially in the group of elderly patients.

## Introduction

The life expectancy of the world's population is continuously increasing. According to the latest WHO report from 2018, the global life expectancy of a child born in 2016 was 70 years for males and 74 years for females [1], a marked increase compared to 2000 where the life expectancy was 64 years for males and 68 years for females [2]. Since the incidence of benign brain tumors such as meningiomas and schwannomas increases with age [3], the medical care systems are facing an increasing number of older patients who suffer from these tumors. Concerning higher co-morbidity rates, treatment concepts in older patients should aim at balancing tumor control against procedural risk.

In addition to micro-neurosurgery, stereotactic radiosurgery (SRS) has evolved as a generally accepted effective therapeutic option, especially for meningiomas and schwannomas. Meanwhile, several studies have reported data on long-term follow-up with tumor control rates exceeding 90% and associated mild toxicity [4–8]. For patients with locally inoperable tumors or patients deemed ineligible for surgery due to medical reasons, SRS such offers a valuable treatment alternative. However, only limited data are available regarding the efficacy and toxicity of SRS for benign brain tumors in the elderly patients group [9–11]. Therefore, in this study, we investigated the efficacy and toxicity of SRS for the most frequent benign brain tumors in older patients.

## Materials and methods

### Patients and subjects

In this single center retrospective analysis, we included patients suffering from either schwannoma or meningioma of grade WHO I who were, or above 65 years of age and were treated with SRS either by means of a modified linear accelerator (LINAC) or by robotic radiosurgery with the Cyberknife^®^ system (CK) within a defined period ranging from January 1991 to March 2018. Patients with neurofibromatosis were excluded. Indications for treatment included all patients with symptomatic or progressive tumors (generally ≤ 3 cm in diameter) that had either tumors rated locally inoperable or bearing a high surgical risk for permanent neurologic deficits; residual or recurrent tumors after previous surgery; refused surgery, or increased risk of surgery due to co-morbidity. In general, treatment decisions were made by an interdisciplinary tumor conference based on these issues.

All relevant patient and treatment data were retrieved from analog and digital patient files. These included tumor entity, age at treatment, gender, indication for treatment, comorbidities, previous treatments for the tumor under investigation, pre-therapeutic symptoms, evolution of symptoms during follow up, tumor volume and irradiation parameters. To evaluate the toxicity of the treatment, early (< 6 weeks after SRS) and late (> 6 months after SRS) signs of toxicity were recorded. This retrospective analysis was approved by the local ethics committee (Reference No. 16-476).

### Treatment planning and SRS procedure

Since 1991, two modified linear accelerators (Philips SL 75/20 at 9MV and Elekta Sli 25 at 6 MV, Elekta, Crawley, UK) were used to administer SRS. These devices were replaced by the Cyberknife^®^ system (Accuray Inc., Sunnyvale, California, USA) in 2012. For treatment planning, either the software STP (STP 3.3 and 3.5, Howmedica Leibinger, Freiburg, Germany) or the CK planning system Multiplan v4.5 was used. From 1991 to 1995, the tumor and the adjacent critical structures (e.g. brainstem, cerebellum, cranial nerves) were outlined on the planning CT (contrast-enhanced, 1 mm slice thickness) by a neurosurgeon experienced in stereotactic radiosurgery. From 1996 onwards, the tumors were outlined on MRI scans (MRI scanner 1.5 or 3 T; Philips, Hamburg, Germany) using a standardized MRI protocol with contrast-enhanced T1-weighted and native T2-weighted MRI sequences which were registered to the planning CT. A treatment plan was generated by a medical physicist and authorized by an interdisciplinary consensus between the stereotactic neurosurgeon, a radiation oncologist also experienced in SRS, and the medical physicist. The planning and dose prescription for meningeoma was based on previous evidence from class III studies [12]. In case of vestibular schwannoma, the IRSA guidelines of 2006 [13] and ISRS guidelines of 2017 [14] were used.

**Fig. 1:**
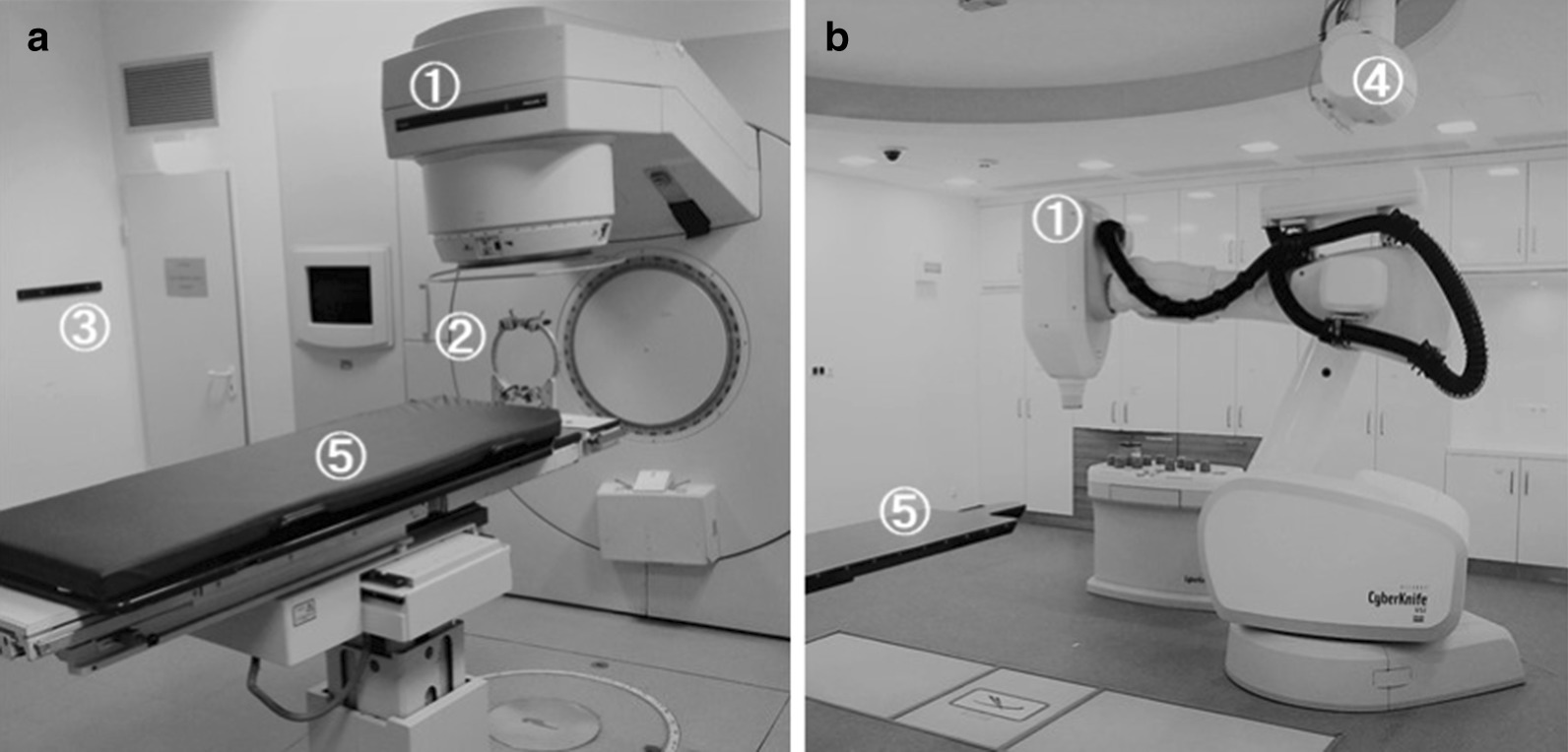
**a** Comparison of the LINAC (**a**) and the Cyberknife (**b**) setting. The relevant components are marked with numbers: (1) linear accelerator (2) stereotactic frame used in LINAC SRS for head fixation. (3) positioning laser (4) X-ray camera (5) mobile patient couch

**Fig. 2:**
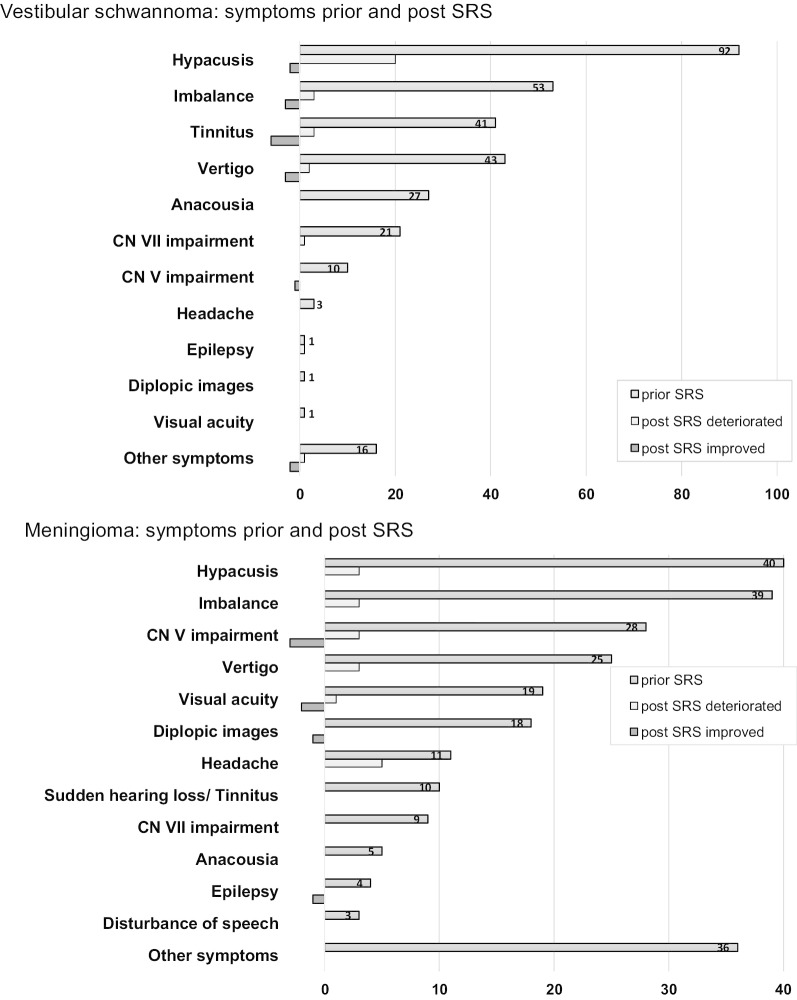
Comparison of symptoms preexisting (prior) to and following SRS in the case of VS and meningioma patients

For radiosurgical treatment with the LINAC, the patient’s head was immobilized under local anesthesia with a stereotactic frame (Riechert-Mundinger) and the radiosurgical treatment was performed using a linear accelerator as previously described [15] and depicted in Fig. 1a. For the CK treatment, the patient was comfortably immobilized on the CK treatment table (Fig. 2b) with a custom-made aquaplast mask. In both types of radiosurgery, peri-interventional cortisone was routinely applied.

### Follow-up evaluation

Follow-up (FU) was routinely scheduled at 6 and 12 months after treatment, followed by annual controls. A distinction was made between the evaluation of clinical and radiological follow-up. Radiologic FU was carried out by contrast- enhanced T1-weighted MRIs. To determine radiologic tumor control (rTC), the largest axial tumor diameter in the anteroposterior and lateral extension on T1-weighted MRIs were measured. A volumetric FU was not feasible because a considerable proportion of the MRIs before 2008 were only available on paper print. Tumor progression was defined as an increase of more than 3 mm in any of the diameters, as according to Hsu et al. [16]. All other tumor changes were defined as rTC. Clinical tumor control (cTC) was assumed by the absence of any further need for treatment [17]. Overall local tumor control (TC) was assumed in the case of both radiological and clinical local tumor control.

At each clinical follow-up (cFU), the development of symptoms was classified either as (1) symptoms improved or symptom-free, (2) deterioration, or (3) newly developed symptoms. Toxicity of SRS was assumed when new, permanent, objectified symptoms developed after SRS. Symptoms were classified according to the Common Terminology Criteria for Adverse Events (CTCAE v 4.03, 2010) and graded as follows: grade 1 (mild symptoms, asymptomatic, or mild symptoms without impact on daily life), grade 2 [moderate, minimal, local or noninvasive intervention indicated; limiting age-appropriate instrumental (ADL)], grade 3 (severe or medically significant, but not immediately life-threatening; hospitalization or prolongation of hospitalization indicated; disabling; limiting self-care ADL), grade 4 (life-threatening consequences; urgent intervention indicated), or grade 5 (death related to AE). In addition, any occurrence of treatment-induced edema and radiation necrosis diagnosed by follow-up imaging was recorded.

### Statistical analysis

All statistical analyses were performed with SPSS, version 25 (IBM Corp., Armonk, New York, USA). Patient data was analyzed using Kaplan–Meier, LogRank test for categorical variables and univariate Cox proportional hazards models for metric variables in terms of overall tumor control (TC), development of edema following SRS and freedom from early and late treatment related complications graded by CTCAE. For both endpoints, data were censored at the time of last FU. A multivariate analysis was not feasible if there were less than ten events per one factor [18]. The following factors were analyzed for possible influence: age (> 70 vs. ≤ 70), gender, multimorbidity (2/ ≥ 3 co-morbidities), malignancy, irradiation system (LINAC vs. Cyberknife), tumor volume, tumor entity (vestibular schwannoma vs. meningioma), Radiation dose, prescription isodose and SRS for recurrence after pretreatment. The presence and severity of symptoms before treatment and up to the last FU was reported by descriptive statistics.

## Results

### Patient collective

In our database, we identified 245 patients aged at least 65 years old suffering from meningioma (M) or vestibular schwannoma (VS) (Table 1). The median age of the patients was 71 years and not significant different between M and VS group (*p* value = 0.75). Of the 245 tumors, 116 (47.3%) were vestibular schwannomas and 129 (52.7%) were meningiomas. We observed 171 patients (69.8%) with co-morbidities, of which 30 were multimorbid (> 2 diseases, 12.2%), 97 had vascular disease (39.6%), 54 had a metabolic disease such as diabetes (22%) and 25 patients (10.2%) suffered from a malignant disease. The indications for SRS were asymptomatic tumor growth in 133 cases (54.3%), tumor remnants after resection in 6 cases (2.4%) and symptomatic tumors in 105 cases (42.9%). In one case, an asymptomatic stable tumor was irradiated due to request by the patient. 56 tumors (22.3%) were recurrent after previous treatment (surgery: n = 51; surgery and radiotherapy: n = 5).

**Table 1:** Clinical characteristics and treatment of patients, Tumor volume, marginal dose and prescribed isodose were statistically significant different between the VS and M group

Patient characteristics	
Patients	245
Gender (m:f)	81:164
Age (years)	
Median (range)	71 (65–86)
Mean (SD)	71.8 ± 5.2
Vestibular schwannoma	116 (47.3%)
Meningioma	129 (52.7%)

Pre-therapeutic comorbidities	
Patients with any comorbidities	171 (69.8%)
Multimorbid patients (≥ 2 diseases)	30 (12.2%)
Comorbidity of vascular disease	97 (39.66%)
Comorbidity of metabolic disease	54 (22%)
Comorbidity of malignant tumor	25 (10.2%)

	Vestibular schwannoma (VS)	Meningioma (M)	*p* value
*Tumor characteristics*			
Treatment indication			
Tumor growth	53 (45.7%)	80 (62%)	
Residual tumor after resection	1 (0.9%)	5(3.9%)	
Symptoms	62 (53.4%)	43 (33.3%)	
Inquiry of patient	–	1 (0.8%)	
Pretreated tumors	20 (17.2%)	42 (32.6%)	
Pretreatment with surgery	20 (100%)	37 (88%)	
Pretreatment with surgery + radiotherapy	–	5 (12%)	
Radiological and clinical follow up available	94 (81%)	114 (89%)	
Radiological follow up (months), mean (SD)	45.5 ± 41.2	40 ± 36.6	0.35
Median (range)	30 (2–173)	26 (4–181)	
≥ 60 months to last radiological FU	25 (26.6%)	28 (24%)	
Tumor volume (cm^3^)			< 0.0001
Mean (SD)	2.7 ± 3.2	5.8 ± 4.6	
Median (range)	1.6 (0.1–23.7)	4.8 (0.2–28)	
*Radiosurgery parameters*			
LINAC (1991–2012), number of patients	99 (85.3%)	73 (56.6%)	
CK (2013–2015), number of patients	17 (14.7%)	56 (43.4%)	
Marginal dose (Gy), mean (SD)	12.3 ± 0.7	12.5 ± 1.2	< 0.002
Median (range)	12 (11–15)	12 (7–20)	
Dose prescription isodose (%), mean (SD)	68.3 ± 12.7	74 ± 7.6	< 0.0001
Median (range)	70 (33.4–85.1)	78 (36–80)	

### Radiation parameters

In total, 172 patients (70.2%) were treated with LINAC (1991–2012) and 73 patients (29.8%) were treated with CK (2013–2018). The median tumor volume was 2.9 ccm (range 0.1–28 ccm). The median marginal dose was mean 12 Gy (range 7–20 Gy). The mean prescription dose (isodose) was 71.3% ± 10.7 (range 33.4–85.1%, median 75%) (Table 1).

### Radiological follow-up

Radiological follow-up was available in 209 patients (85.3%), of whom 25% (n = 53) were observed for ≥ 60 months. The mean and median radiological FU interval was 42 months and 28 months (range 2–181 months), respectively. Among the VS patients radiological progression occurred in 2 cases (1.7%). In the meningioma patients, loss of TC due to progression was observed in 8 cases (6.2%).

One VS patient (0.6%) developed a symptomatic edema after six months. In the meningioma group, edema occurred in 13 patients (10%) and radiogenic tumor necrosis in one patient (0.7%). Six of these edemas were symptomatic. The mean time interval between SRS and peritumoral changes for meningioma was 11.4 months (range 3–28 months). Of these 15 morphologic changes, 6 were classified as permanent because they lasted until last follow-up. Univariate cox regression analysis revealed a significant impact of tumor volume (*p* = 0.015 CI-95 1.02–1.2, HR 1.1) on development of edema. All other variables tested were not significant.

### Clinical follow-up and toxicity

Clinical and radiological follow-up intervals were similar (Table 1). Schwannomas were significantly more symptomatic (Fig. 2).

Immediately after treatment, a total of four patients developed new transient disorders. One VS patient experienced a mild facial paralysis. The other three meningioma patients experienced neuropathy of the trigeminal nerve (n = 1), new onset of headaches (n = 2), and vertigo (n = 1). The pre- and post-therapeutic symptoms are shown in Fig. 2.

Permanent adverse events that were objectified in terms of the CTCAE had a crude rate of 5.7% (n = 14/245) comprising five patients with CTCAE grade 1 (3.9%), six patients with CTCAE grade 2 (4.6%), and 3 patients with CTCAE grade 3 (2.3%) (Table 2).

**Table 2:** Incidence of permanent CTCAE-classified adverse events of either meningioma or VS patients (number of patients shown)

	Meningioma (M)					
	< 6 mo			> 6 mo		
	Grade 1	Grade 2	Grade 3	Grade 1	Grade 2	Grade 3
Tinnitus	1					
Vertigo	2				1	1
Headache	1					
Epilepsy			1			
CN XII impairment	1					
Paresis		1				

	Vestibular schwannoma (VS)					
Vertigo and tinnitus					1	
Headache		1			1	
CN VII impairment			1		1	

**Fig. 3:**
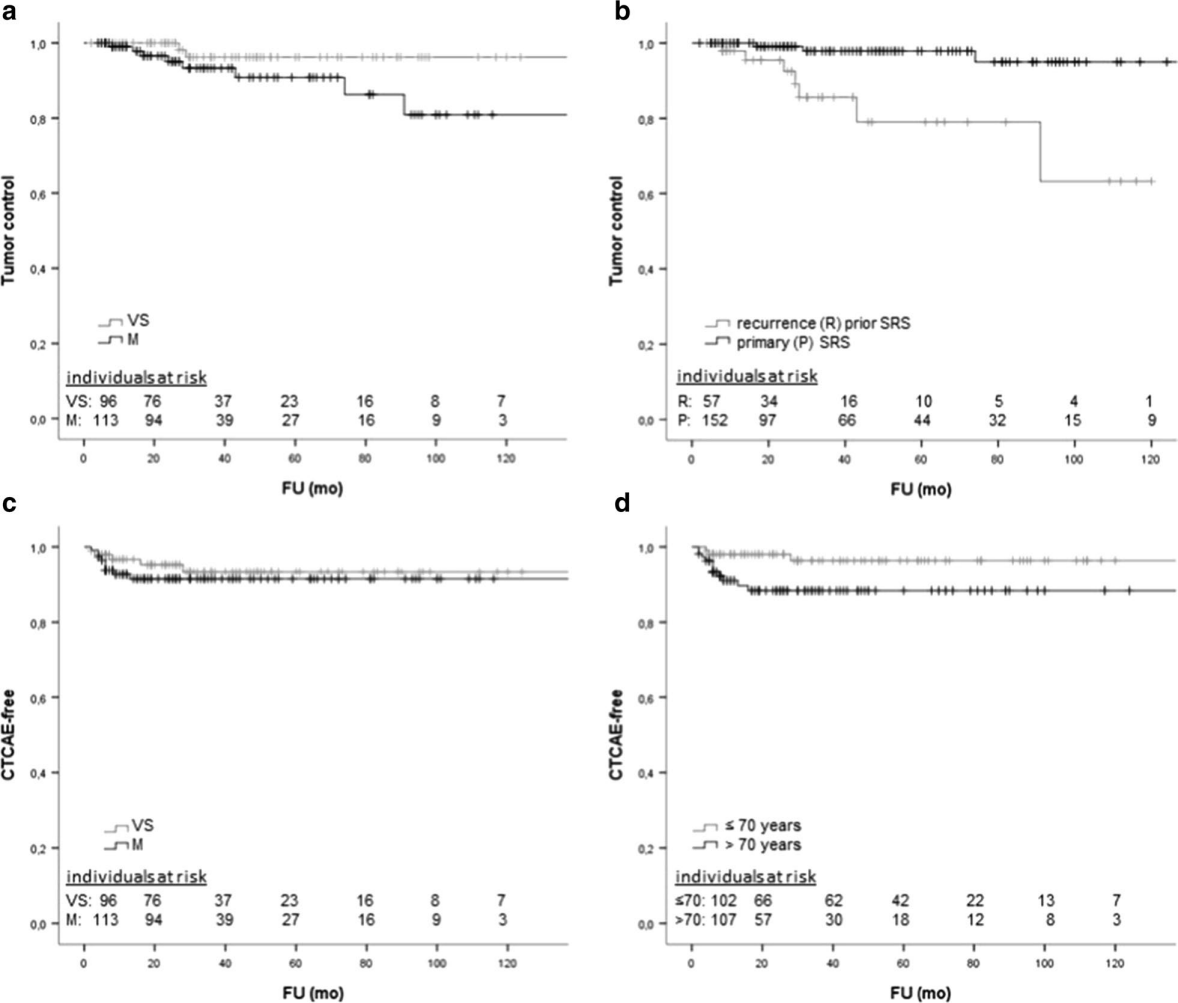
Comparison of tumor control and CTCAE-free status. **a** Comparison of overall tumor control after SRS of VS and meningioma in elderly patients (> 65 years). Actuarial tumor control for meningioma was 96%, 91% and 81% and for VS 100%, 96% and 96% after 2, 5 and 10 years, respectively. No statistical significant difference (*p* = 0.079) was observed between the two tumor groups. **b** Comparison of overall tumor control after SRS in patients with SRS treatment of recurrent tumors and primary SRS. Patients with recurrence prior SRS had a significantly higher risk for tumor progression (*p* < 0.001). c Comparison of CTCAE-free status after SRS. No statistical significant difference (*p* = 0.079) was observed between the two tumor entities. **d** Comparison of CTCAE-free status after SRS with regard to age ≤ 70 and > 70 years. Patients exceeding 70 years of age had a significantly higher risk for toxicity (*p* = 0.027)

The majority of the events occurred within the first six months after irradiation. Kaplan–Meier analysis revealed a CTCAE-free status after 2 years in 93%, and after 5 and 10 years in 92% of the patients (Fig. 2c). Advanced age (> 70) was a prognostic factor for shorter CTCAE-free status (LogRank *p* = 0.027, Table 3, Fig. 3d).

**Table 3:** Prognostic factors in elderly patients with benign meningioma or VS treated with SRS

Factors	Tumor control	Freedom from toxicity (CTCAE)
	*P* value	*P* value
Age (> 70 vs. ≤ 70)	0.106	0.027^a^
Gender	0.431	0.149
Multimorbidity (≥ 2 co-morbidities)	0.248	0.166
Malignancy as co-morbidity	0.213	0.213
Irradiation system (LINAC vs. CK)	0.141	0.051
Tumor volume	0.080	0.056
Entity (VS vs. meningioma)	0.079	0.079
Radiation dose	0.324	0.753
Prescription isodose	0.261	0.200
Recurrence after pretreatment	< 0.001^a^	0.471

^a^*P* values < 0.05 were considered significant

### Local tumor control

The crude rate of tumor progression (loss of rTC) after SRS was 4.8% (n = 10 out of 209 with radiological follow-up). Salvage therapies were applied to eight of these. Six recurrent tumors were surgically resected and SRS was re-applied for two tumors. The other two patients refused further treatment. Since rTC corresponded to cTC, the crude rate of overall tumor control (TC) was almost 96% over the whole FU period. The actuarial TC after 2, 5 and 10 years was 98%, 93% and 88%, respectively (Fig. 3a). The TC of VS and M did not differ significantly (*p* = 0.079, Table 3) and is given in detail in the legend of Fig. 3a. Recurrent tumors seem to pose a higher risk for local control failure of SRS (LogRank *p* < 0.001, Table 3, Fig. 3b). Comorbidities (LogRank, *p* = 0.497), malignancies (LogRank, *p* = 0.213) and multimorbidity (LogRank, *p* = 0.166) had no significant influence on local tumor control.

## Discussion

For the growing cohort of elderly patients with considerable co-morbidities, treatment concepts balancing tumor control against risk are needed in cases of failed “wait and scan” strategy. In this particular context, stereotactic radiosurgery may result in high tumor control with low toxicity rates.

The number of reported SRS series for elderly patients (> 65 years) is astonishingly low. Although a literature query of the NCBI database using the headings "radiosurgery elderly vestibular schwannoma" OR "radiosurgery elderly meningioma" FOR the last decade revealed more than 620 results, only the study by Hasegawa et al. [10] reported single fraction radiosurgery of meningiomas in an elderly cohort (> 65 years). Two other series report SRS with different fractionation concepts [9, 11]. For vestibular schwannoma, no study with special regard to elderly patients is available. Therefore, this present study is the first to report a detailed analysis of the treatment of SRS for this subpopulation.

### Tumor control

Our study confirms the high rate of tumor control (> 90%) and effectiveness of SRS, as reported in many other series [7, 8, 19, 20]. However, the influence of age on tumor control in benign brain tumors is currently not fully understood. Although several studies demonstrated no impact of age on tumor control, others suggest age as a risk factor for tumor recurrence [5, 21]. Although a clear cut-off value for age in general not seem to exist, Starke et al. report about 65 years as a higher risk of local control failure [21]. Since the anti-proliferative effect of radiosurgery for benign tumors depends not only on cytotoxic but also on delayed vascular effects [22], one might speculate that these processes are less effective in older patients. If so, one might assume that patients with vascular diseases have a higher risk for recurrence. However, our results do not support this hypothesis. The important conclusion of our finding is that patients over 70 years of age do not have a higher risk for local control failure.

Patients with recurrence after previous treatment (surgery in most of the cases) had a higher risk for local control failure after SRS. This is in accordance with a study by Hasegawa et al. [19], suggesting higher aggressiveness in recurrent tumors. Similar results for meningiomas [23, 24], as reported e.g. by Kim et al. [23] in more than 700 patients, explained the higher local recurrence rate after previous microsurgery by a surgery-related breakdown of the stroma capsule, rendering radiosurgery less efficient. Furthermore, after previous treatment scarring might arise, which hinders defining the exact target volume of SRS, especially in situations of dural insertion of the tumor. Therefore, further studies in a larger collective may help to elucidate the patterns of failure. However, a particular reason for treatment failure of radiosurgery after previous surgery remains to be identified.

Overall, the present local tumor control rates (93% at 5 years) are in the upper range compared to those observed in other series of elderly patients [10]. In particular, these results were obtained in a distinctive collective with 50% treatments primarily due to proven tumor growth prior to SRS. During follow-up, approximately 1/3 of all tumors decreased measurably in diameter during the observation period. Thus, SRS provided control of tumor growth for the majority of patients in our series, but did not provide rapid tumor shrinkage. If the latter is necessary for symptom alleviation, surgical removal is mandatory [25, 26].

On the other hand, microsurgery bears relevant risks for elderly patients, particularly in the presence of severe and/or multiple co-morbidities, even in a situation of a space-occupying, symptomatic lesion. Therefore, if the primary treatment goal is the mere control of tumor growth, surgery should be weighed against SRS and radiotherapy. In a large series reported by Sughrue et al. [27], the 5-year recurrence rate after resection of WHO I meningioma (n = 373) for patients receiving a Simpson Grade I, II, III, or IV resection was 95, 85, 88, and 81%, respectively. The authors concluded that a Simpson Grade I resection is beneficial if it is easily obtained with a low risk. But the primary goal of meningioma surgery should be to remove as much tumor as possible, e.g. to reduce pressure. In cases where there is an increased risk of neurological or vascular injury, or CSF leak, the authors found it hard to justify performing more aggressive attempts of resection only to improve the rate of recurrence by a few percent, even if the recurrence rates match the rates of SRS.

### Toxicity

When analyzing toxicity, we included any symptom occurring after treatment, without regarding any causal relationship, and classified it according to the CTCAE classification. Some of these symptoms may have been caused by the tumor itself or could have developed anyway. Thus, our results might overestimate the risk of toxicity to a certain amount. In addition, symptoms that occurred immediately after treatment were reversible in total.

In contrast to these low toxicity rates observed after SRS, surgical treatments are often associated with higher complication rates. In a current meta-analysis of Poon et al. [28], a general complication rate of 20% was observed for surgical resection of meningioma in elderly people. Furthermore, it has also been shown that after resection of benign brain tumors, older patients have higher hospital mortality rates and longer hospital stays than younger patients [29], and one-year mortality rates in these elderly patients may reach 15% [30, 31].

The assessment of treatment-related imaging changes such as edema and radiation necrosis can help to objectify the toxicity of SRS. In a current review by Milano et al. [32] the frequency of radiation-induced edema was reported to amount to between 2% up to 50%. We observed a favorable low crude rate of edema formation nearly similar to current observation studies [33]. The causes for edema formation are discussed widely and clear relationships to potential risk factors such as tumor volume, radiation dose, previous treatment with radiation, location of the tumor, presence of edema before treatment or extent of tumor-brain were identified. Unger et al. [34] considered a large tumor volume and single-fraction irradiation as main risk factors for edema formation after treatment. In the case of tumor volume, our results confirm the findings in literature [33, 34].

In conclusion, our findings suggest that elderly patients with larger tumors may have an increased risk for edema development after SRS but most of these imaging changes remain symptomless. According to Chin et al. [35], radionecrosis (RN) is the most important complication of SRS and it depends on tumor volume, 10-Gy volume [36] and re-irradiation of the same tumor, and shows an onset time of about four months. Compared to the reported RN frequencies ranging from 2 to 25% [35, 37], the incidence of RN in our study (about 0.5%) is extremely low. One reason might be the moderate size of tumor volumes treated in our cohort.

Finally, an important finding of our study is that patients' co-morbidities have no influence on the effectiveness and especially the toxicity of the treatment, whereas for surgery it is always an issue. A recent review of meningioma surgery in elderly patients [38] often found that postoperative mortality is most commonly associated with co-morbidities. Eksi et al. [39] also found in their meta-analysis that co-morbidities are a strong predictor of postsurgical neurologic complications. Especially in the group of elderly patients, these aspects should be considered, while taking into account the results of tumor control after SRS presented here. Thus, it is worth considering SRS as primary treatment of meningioma and schwannoma in the group of elderly patients even if they have severe co-morbidities.

### Limitations of our study

Due to the retrospective nature, follow-up times are limited in our study. The reasons for this was lacking compliance, long travelling distances and changes in residence location preventing patients from attending follow-up at the referring hospital. Furthermore, the study is based on a heterogeneous cohort with potential bias induced by large divergences in premorbid factors, but perhaps this collective best reflects daily clinical practice.

## Conclusion

SRS using LINAC or Cyberknife results in favorable overall outcome and reliable tumor control in elderly patients. SRS is therefore a safe and effective treatment option for this cohort, independently of pre-existing co-morbidities. However, this study implies that tumor growth from a previous resection site plays an important role, as well as the patient's age > 70 years.

## Data Availability

All data generated or analysed during this study are included in this published article. The individual datasets of each patient generated during and/or analysed during the current study are not publicly available because individual privacy could be compromised but data are available from the corresponding author on reasonable request.
